# Epidemiological dynamics and rising trends of MRSA in Saudi Arabia: a 12-year observational study

**DOI:** 10.3389/fcimb.2025.1622647

**Published:** 2025-10-08

**Authors:** Anandhalakshmi Subramanian, Yahya Shabi, Tarik Alazraqi, Ihab M. Abdelrahim, Mohamed E. Hamid, Ali Al Bshabshe, Abdullah Algarni, Sara Habbash, Safia Abdullah Mohammed, Abdulah Jarboa Alqahtani, Rasha Elsherif, Abdulah O. S. Bawazeer, Saeed M. S. Alhamhhum, Khalifa Binkhamis

**Affiliations:** ^1^ Department of Microbiology and Clinical Parasitology, College of Medicine, King Khalid University, Abha, Saudi Arabia; ^2^ Department of Medicine, Aseer Central Hospital, Abha, Saudi Arabia; ^3^ Department of Medicine/Adult Critical Care, College of Medicine, King Khalid University, Abha, Saudi Arabia; ^4^ Department of Family Medicine, Aseer Central Hospital, Abha, Saudi Arabia; ^5^ Clinical and Chemical Pathology Department, Faculty of Medicine, Cairo University, Cairo, Egypt; ^6^ Department of Pathology, Aseer Regional Lab, Abha, Saudi Arabia; ^7^ Department of Microbiology, Aseer Central Hospital, Abha, Saudi Arabia; ^8^ Department of Pathology, College of Medicine, King Saud University, Riyadh, Saudi Arabia

**Keywords:** MRSA, MSSA, *mecA*, SCCmec gene, Saudi Arabia, epidemiology, hospital-associated infections, longitudinal study

## Abstract

**Background:**

Methicillin-resistant *Staphylococcus aureus* (MRSA) presents serious clinical and public health complications, and *Staphylococcus aureus* is still a major cause of morbidity and mortality globally. The objective of this study was to assess the temporal dynamics, microbiological traits, and epidemiological trends of methicillin-susceptible *S. aureus* (MSSA) and MRSA isolates over a twelve-year period in a tertiary care facility.

**Methods:**

This retrospective cohort study analyzed the data of all confirmed *S. aureus* isolates collected between January 2013 and June 2024. Identification and antimicrobial susceptibility testing were performed using automated methods according to CLSI guidelines. MRSA was confirmed by detection of the *mecA* gene in all isolates using the GeneXpert MRSA assay. Additionally, 100 randomly selected MRSA isolates were further tested with the same platform for the presence of the SCC*mec* gene; all of which were positive. Demographic, clinical, and microbiological data were evaluated, and Generalized Linear Models were applied to assess temporal trends in oxacillin resistance.

**Results:**

The total of confirmed *S. aureus* isolates was 4,267. MRSA accounted for 52.7% (2,250) of all *S. aureus* isolates. It was significantly more prevalent in patients with COVID-19 (62.5%), diabetes mellitus (56.4%), and end-stage renal disease (52.7%) (p = 0.041). MRSA rates were higher in inpatient settings, particularly in surgical (56.0%), ICU/IMCU (53.4%), and medical wards (52.7%) (p < 0.001). While culture sources did not differ significantly between MRSA and MSSA (p = 0.212), MRSA was more commonly found in blood, skin, and abscess samples. Over time, MRSA prevalence increased across all wards, with the surgical ward showing the most significant rise (OR = 1.115; 95% CI: 1.080–1.152; p < 0.001).

**Conclusion:**

This study demonstrates a rising burden of MRSA over the past decade, especially among vulnerable populations. The findings underscore the need for strengthened infection control, targeted antimicrobial stewardship, and ongoing surveillance to combat MRSA in Saudi Arabia and similar high-risk settings.

## Introduction

*Staphylococcus aureus* is a versatile and opportunistic pathogen implicated in a wide spectrum of infections, ranging from minor skin and soft tissue conditions to severe, invasive diseases such as pneumonia, endocarditis, osteomyelitis, and bloodstream infections (Tong et al., 2015). The global escalation of antimicrobial resistance, particularly the emergence and spread of methicillin-resistant *S. aureus* (MRSA), has transformed the clinical practice, posing significant challenges to treatment, infection control, and public health management (Gurung et al., 2020; Bereanu et al., 2024).

While methicillin-susceptible *S. aureus* (MSSA) remains responsive to beta-lactam antibiotics, MRSA exhibits resistance to multiple drug classes, including beta-lactams, macrolides, and fluoroquinolones (Vestergaard et al., 2019). This multidrug-resistant phenotype has contributed to higher rates of morbidity, mortality, and healthcare costs (Abebe and Birhanu, 2023; Kumar et al., 2025). The emergence of MRSA has led to an increased reliance on last-line agents such as vancomycin and linezolid, further complicating therapeutic decisions and antimicrobial stewardship (Kumar et al., 2025).

A meta-analysis from Egypt reported a pooled MRSA prevalence of approximately 67% among *Staphylococcus aureus* clinical isolates, mainly collected from hospitals and healthcare facilities, including blood, respiratory specimens, wound swabs, and urine (Azzam et al., 2023). In a systematic review and meta-analysis of 119 studies from 29 countries, the pooled global MRSA prevalence of 14.7% (95% CI: 12.4–17.2%) among residents of elderly care centers. This study emphasized the high prevalence of MRSA in these settings and the need for targeted infection control measures (Hasanpour et al., 2023).A systematic review and meta-analysis of 40 studies across 20 countries reported a global MRSA prevalence of 17% (95% CI: 14–27%) in diabetic foot ulcer patients. The prevalence declined from 25% before 2010 to 9% after 2021. Regionally, it was highest in South America (61%), followed by North America (20%), Europe (19%), and Africa (13%) (Zhou et al., 2024). In China, the prevalence of MRSA has shown a notable decline over recent years. A study published in Scientific Reports reported a decrease in the proportion of MRSA from 69% in 2005 to 44.6% in 2014 (Zhen et al., 2020).

In Saudi Arabia, the epidemiology of MRSA has demonstrated considerable regional variability. National estimates suggest that MRSA accounts for approximately 35% to 45% of all *S. aureus* isolates, with higher rates reported in hospital settings (El Amin and Faidah, 2012; Alanzi et al., 2024; Almutairi et al., 2024). A systematic review covering data from 2002 to 2012 documented an overall MRSA prevalence of 35.6% (Yousef et al., 2013). Regional variations are notable. A 2019 study indicated that the Western region, which includes the Asir region, had a MRSA prevalence rate of 42%, higher than the Central (32%) and Eastern (27%) regions (Adam and Abomughaid, 2018). Studies from Riyadh have reported that 27% to 33% of *Staphylococcus aureus* isolates were MRSA (Baddour et al., 2006), while research from Jeddah found the rate to be around 40% (Madani, 2002).

MRSA is particularly prevalent in hospital-associated infections (HAIs), especially in intensive care units (ICUs), where rates have been reported as high as 60% to 80% (Madani, 2002; Ali et al., 2020). In contrast, MSSA remains more prevalent in community-acquired infections and is a significant contributor to *S. aureus* disease burden. MSSA is typically associated with lower resistance profiles and better treatment outcomes. However, its proportion among *S. aureus* isolates has gradually declined in many regions due to the rise of MRSA. In the Asir region, the dominance of MRSA in hospital settings underscores an urgent need for targeted infection control and surveillance efforts (Hamid, 2011). A study reported that hospital-acquired MRSA represents 54% of all nosocomial infections caused by *S. aureus* clinical isolates in the Asir Province, with 85% of these strains being multidrug-resistant (Alanzi et al., 2024).

According to a recent systematic analysis conducted in 2024, which included more than 16,000 samples from 24 studies across the Kingdom, the average MRSA prevalence was 17.0% (random effects) and 8.6% (fixed effects).

These studies demonstrate the importance of having local epidemiology data to understand resistance patterns and guide strategic interventions. Given the increasing incidence of MRSA in both healthcare and community environments, ongoing surveillance is critical (Samuel et al., 2023). Monitoring temporal resistance patterns enables clinicians and public health officials to adapt empiric therapy protocols, reinforce antimicrobial stewardship, and develop regionally appropriate guidelines for infection prevention and control (Pezzani et al., 2020).

This study aimed to describe the epidemiological and microbiological characteristics of *S. aureus* infections, focusing on the comparative prevalence of MRSA and MSSA isolates across different clinical settings and comorbidities. We also investigated the trends in oxacillin resistance over 11 years (2013–2024), evaluating the temporal progression and the specific healthcare environments where MRSA has become increasingly prevalent. These findings are expected to provide crucial insights that support targeted antimicrobial stewardship and guide infection control policies tailored to regional needs.

## Methodology

### Study design and setting

This retrospective observational study analyzed microbiological data from all non-duplicate clinical isolates of Staphylococcus aureus obtained between January 2013 and June 2024 at the microbiology laboratory of Aseer Central Hospital, a tertiary care facility in Abha, Saudi Arabia. Only the first single isolate per patient per infection episode was included to avoid duplication. Pediatric(<18 years) and neonatal isolates, as well as colonization or surveillance samples (e.g., nasal swabs for MRSA screening), were excluded. The study was conducted under a waiver of informed consent granted by the institutional ethics committee, in accordance with ethical guidelines, ensuring patient confidentiality and data protection.

### Microbiological identification and susceptibility testing

All isolates were identified and tested for antimicrobial susceptibility using automated phenotypic systems (VITEK 2 [bioMérieux, France] or BD Phoenix 100 [Becton Dickinson, USA]. Results were interpreted according to the Clinical and Laboratory Standards Institute (CLSI) performance standards valid during the respective study years (M100 editions 2013–2023). Methicillin resistance was determined based on oxacillin and cefoxitin resistance profiles, the CLSI-recommended reference method for MRSA detection in routine clinical microbiology. Subsequently, all isolates were confirmed by detection of the *mecA* gene using the GeneXpert MRSA assay (Cepheid, USA). This ensured methodological consistency and comparability across the 12-year study period. Further, 100 randomly selected MRSA isolates were tested by GeneXpert for the presence of the staphylococcal cassette chromosome (*SCCmec*) gene. All tested isolates were positive for the *SCCmec* gene. Clinical specimens included blood, respiratory tract samples, urine, wound swabs, abscesses, body fluids, cerebrospinal fluid, and soft tissue collected from both general wards and intensive care units (ICUs).

### Data collection and variables

The source of data was the hospital’s electronic laboratory information system (LIS), and quality control was maintained by internal protocols and CLSI recommendations throughout the study period. While diagnostic platforms and interpretative breakpoints were periodically updated to align with CLSI revisions, the overarching criteria for classifying MRSA and MSSA remained consistent.

The dataset included demographic variables (age, sex), clinical diagnoses, culture sources, and hospitalization details, including ward of admission and inpatient/outpatient status. Comorbidities and risk factors were categorized into long-term stay, End-Stage Renal Disease (ESRD), Burns, COVID-19, Diabetes Mellitus (DM), and others.

### Statistical analysis

Statistical analyses were performed using SPSS version 26.0 (IBM Corp., Armonk, NY, USA). Continuous variables (e.g., age) were expressed as mean ± standard deviation (SD) and compared between MRSA and MSSA groups using independent-samples t-tests. Normality was verified using the Shapiro–Wilk test. Homogeneity of variances was assessed with Levene’s test, and the appropriate p-value (equal variances assumed vs. not assumed) was reported accordingly.

Categorical variables (e.g., sex, ward, comorbidities, and culture source) were analyzed using Pearson’s chi-square test or Fisher’s exact test when expected cell counts were <5. To evaluate temporal changes in MRSA prevalence, Generalized Linear Models (GLM) with a binomial distribution and logit link were applied. Model assumptions and overall fit were checked using deviance and Pearson chi-square statistics. Odds ratios (ORs) with 95% confidence intervals (CIs) were reported to quantify longitudinal trends.

Missing data were minimal (<5% across variables) and were managed using pairwise deletion without imputation. No duplicate isolates from the same patient or infection episode were included in the analysis, ensuring that each case represented a unique isolate. A two-tailed p-value <0.05 was considered statistically significant.

## Results

A total of 4,267 non-duplicate *Staphylococcus aureus* isolates were included in the analysis, comprising 2,250 (52.7%) MRSA and 2,017 (47.3%) MSSA. The mean age of patients was 49.3 ± 22.4 years, with no significant difference observed between the MRSA and MSSA groups (p = 0.175). Males accounted for 70.8% of the study population, and although a slightly higher proportion was noted among MRSA cases (53.3%), the difference was not statistically significant (p = 0.281) (**Table 1**).

**Table 1:** Demographic distribution of MRSA and MSSA.

Characteristic	Total samples	MSSA	MRSA (*mecA*)	P Value
	n = 4267	n = 2017	n = 2250	
Age (mean ± SD)	49.30 ± 22.35	48.81 ± 22.80	49.74± 21.94	0.175a
Sex				0.281b
Male	3023 (70.8%)	1413 (46.7%)	1610 (53.3%)	
Female	1244 (29.2%)	604 (48.6%)	640 (51.4%)	

SD, Standard deviation.

a-independent t-test.

b—Binomial generalized linear model for trend.

MRSA was significantly associated with certain clinical diagnoses, including urinary tract infections (62.5%), COVID-19 (62.5%), diabetes mellitus (56.4%), and ESRD (52.7%) (p = 0.041) (**Table 2** **).**

**Table 2:** Correlation Between Patient Risk Factors and Prevalence of MSSA vs MRSA.

Risk factors	Total samples	MSSA	MRSA(*mecA*)	P Value
Long-term stay	355 (8.3%)	196 (55.2%)	159 (44.8%)	0.030a
ESRD	112 (2.6%)	53 (47.3%)	59 (52.7%)	
Burn	78 (1.8%)	41 (52.6%)	37 (47.4%)	
COVID-19	40 (0.9%)	15 (37.5%)	25 (62.5%)	
DM	39 (0.9%)	17 (43.6%)	22 (56.4%)	
Other	3643 (85.4%)	1695 (46.5%)	1948 (53.5%)	

ESRD, end-stage renal disease.

DM, diabetes milletus.

a Chi-square test.

**Figure 1:**
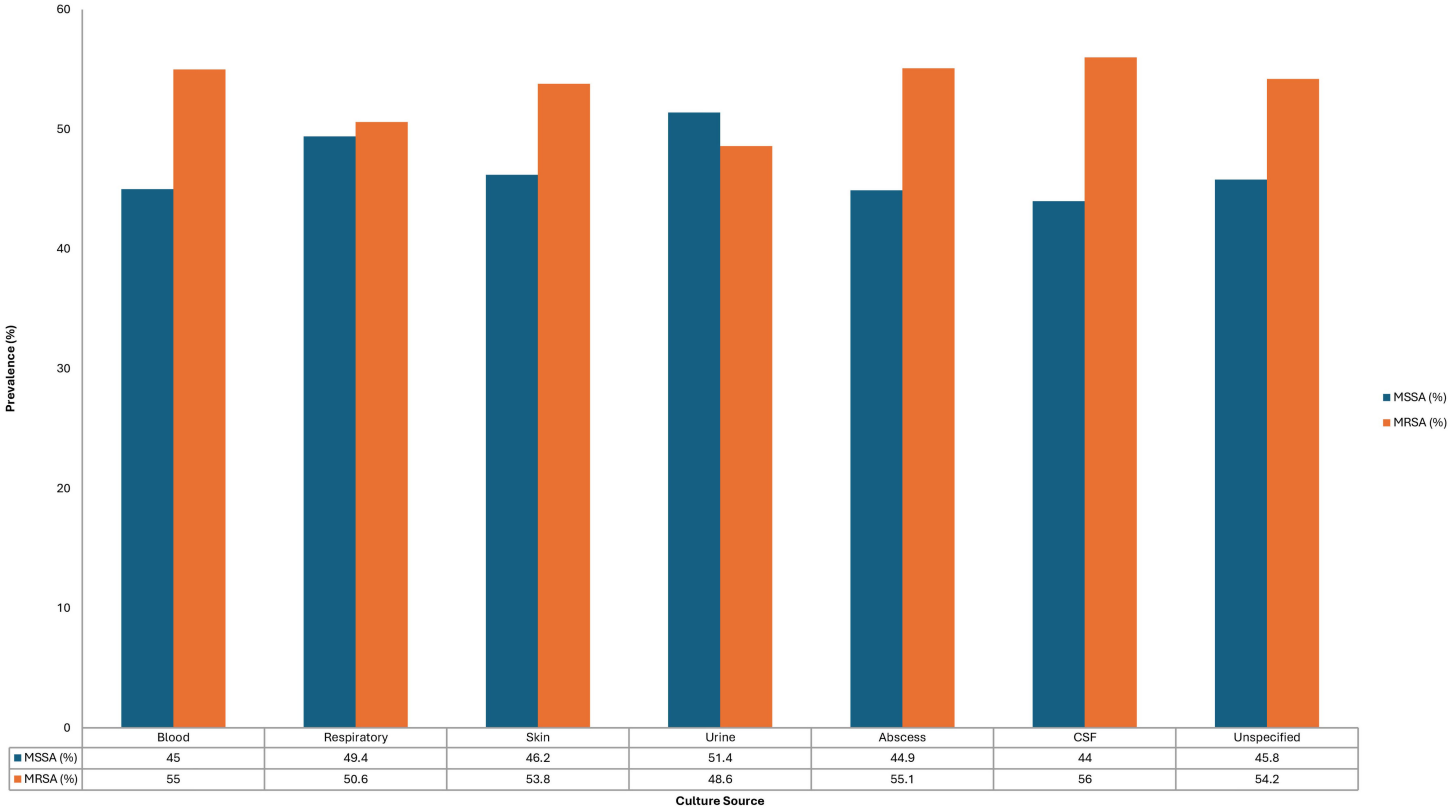
Comparative distribution of MRSA and MSSA isolates across hospital wards.

Inpatient status also correlated with higher MRSA prevalence (54.1%, p < 0.001; Fisher's exact test). Across hospitalization wards, MRSA was more frequently isolated in surgical (56.0%), ICU/IMCU (53.4%), and medical (52.7%) wards compared to MSSA, with statistically significant differences (p < 0.001) (**Figure 1**).

**Figure 2:**
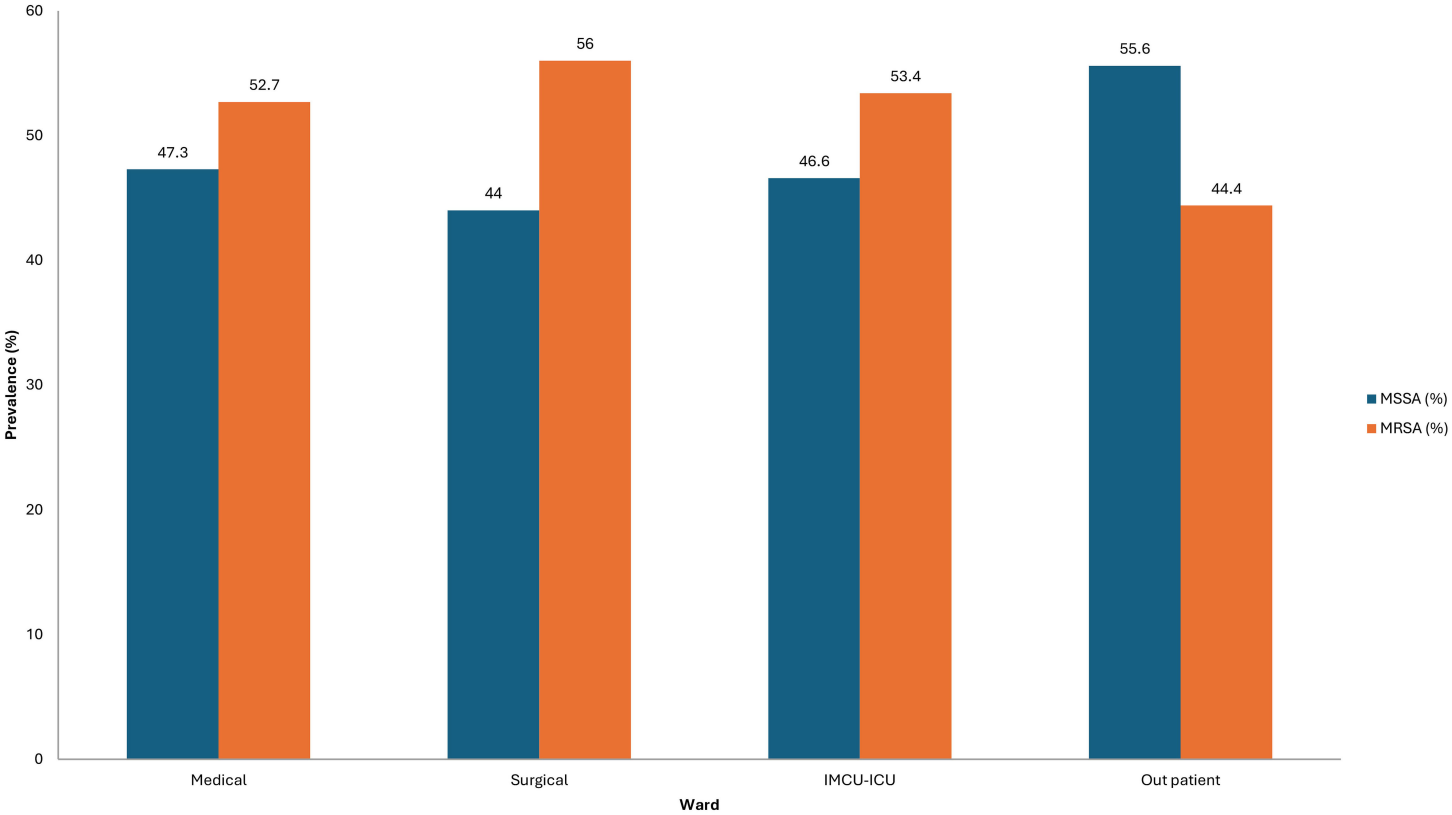
Spectrum of clinical sources for MRSA and MSSA.

The distribution of culture sources did not differ significantly between MRSA and MSSA isolates (p = 0.212); however, MRSA was more frequently recovered from cerebrospinal fluid (56%), blood (55.0%), skin and soft tissue (53.8%), and abscess samples (55.1%) (**Figure 2**).

**Figure 3:**
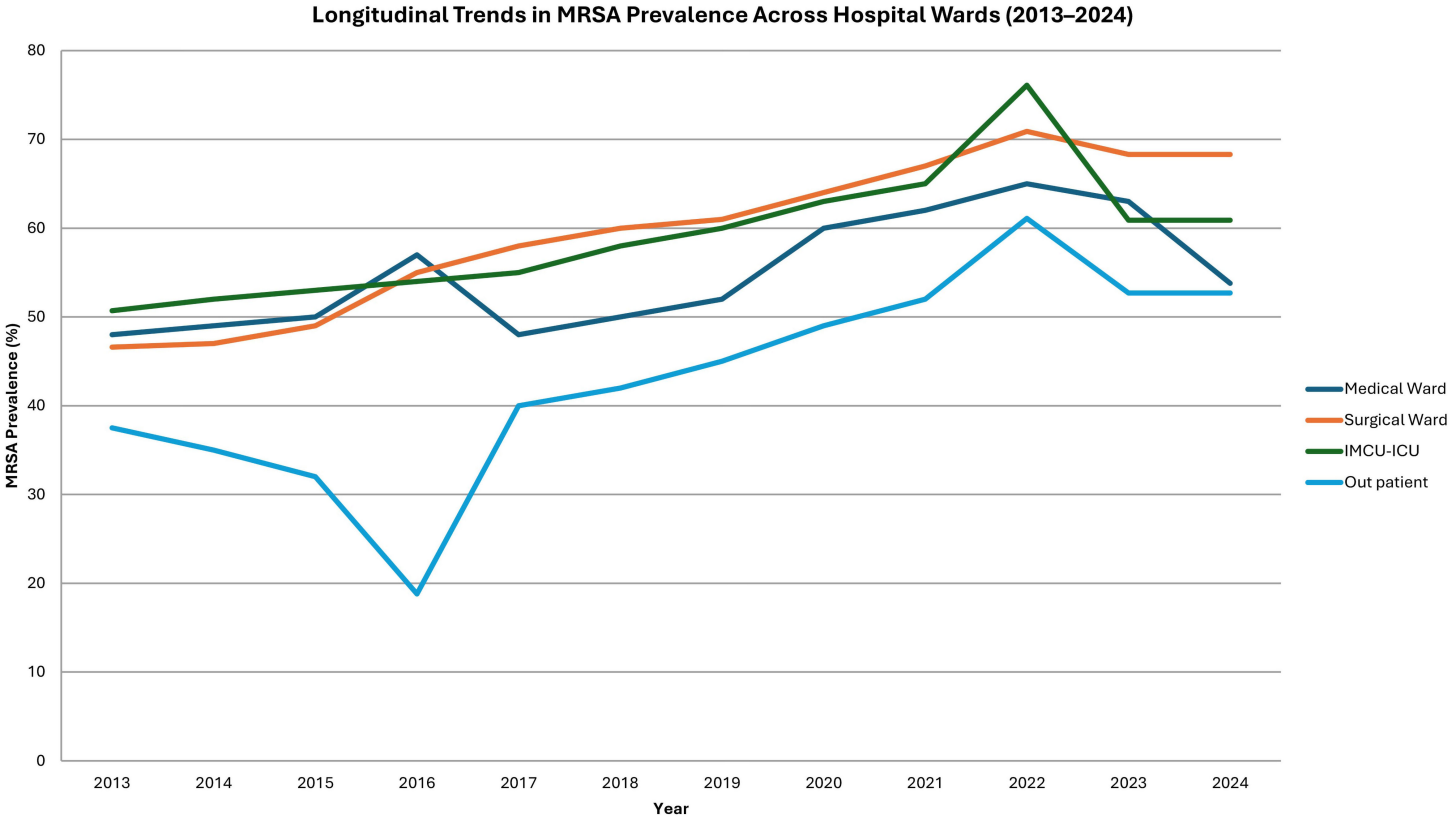
Longitudinal trends in MRSA prevalence across hospital wards (2013–2024).

MRSA prevalence demonstrated a statistically significant upward trend across all hospital wards over the study period. In surgical wards, the proportion of MRSA isolates increased from 46.6% in 2013 to 68.3% in 2024 (OR = 1.115; 95% CI: 1.080–1.152; p < 0.001). Comparable increases were observed in medical wards (OR = 1.066; p < 0.001), IMCU/ICU units (OR = 1.050; p = 0.021), and outpatient settings (OR = 1.112; p < 0.001) (**Figure 3**).

## Discussion

This study presents a 12-year analysis of *Staphylococcus aureus* infections, with particular emphasis on the evolving epidemiology of MRSA in comparison to MSSA within a tertiary care center. The findings demonstrate a sustained and concerning rise in MRSA prevalence, aligning with both national and international epidemiological trends.

In our study, we confirmed all MRSA isolates by detecting the mecA gene with the GeneXpert MRSA assay. We also confirmed 100 randomly selected isolates as positive for the SCC*mec* gene. These findings match global reports, where *mecA* is the main cause of methicillin resistance in *S. aureus* and is nearly always found in MRSA strains. For instance, studies from Europe, Asia, and North America show *mecA* positivity among MRSA isolates (Becker et al., 2016; Idrees et al., 2023; González-Vázquez et al., 2024). The detection of SCC*mec* elements further highlights their role in spreading resistance. Hospital-associated lineages often carry larger SCC*mec* types (I–III), while community-associated strains are usually related to smaller, more mobile types (IV and V) (González-Vázquez et al., 2024).

The overall MRSA prevalence in this study (52.7%) surpasses earlier estimates from Saudi Arabia, where a systematic review reported MRSA rates ranging from 35.6% to 45% in various regions between 2002 and 2012 (El Amin and Faidah, 2012; Alanzi et al., 2024). Our observed rate aligns closely with more recent reports from the Asir region, where MRSA prevalence in nosocomial *S. aureus* infections was noted to be approximately 54% (Hamid, 2011). Similarly, a study conducted in Taif (Mecca Province) reported that nosocomial infections accounted for 48% of all *S. aureus* infections among hospitalized patients, while community-associated infections comprised 52% (Sabra and Abdel-Fattah, 2012). This suggests that the burden of MRSA in the Asir region may be higher than the national average, which reflects a broader trend of increasing resistance, particularly in hospital settings.

Globally, MRSA prevalence varies widely, from 20%–30% in northern Europe to over 50% in parts of Asia and the Middle East (World Health Organization, 2014). Studies from neighboring Gulf countries, such as the United Arab Emirates and Kuwait, report MRSA prevalence rates between 22% and 55%, indicating that Saudi Arabia’s MRSA burden is comparable to regional figures (Udo and Boswihi, 2017; Al-Saleh et al., 2022; Thomsen et al., 2023).

Importantly, our study demonstrated no significant differences in MRSA prevalence by age or sex, consistent with findings from other studies in Saudi Arabia and internationally (Madani et al., 2001; Iyer et al., 2014). However, MRSA infections were significantly associated with specific risk factors, particularly COVID-19 (62.5%), diabetes mellitus (56.4%), and end-stage renal disease (52.7%) (p = 0.030). Similar associations have been noted in global studies, where MRSA is increasingly implicated in infections among immunocompromised and chronically ill patients (Zacharioudakis et al., 2014; Garoy et al., 2019; Samuel et al., 2023).

Hospitalization settings significantly influenced the prevalence of MRSA. According to our findings, the percentage of MRSA in inpatients was substantially higher (54.1%, p < 0.001), especially in surgical wards (56.0%), ICU/IMCU units (53.4%), and medical wards (52.7%). This is consistent with previous Saudi research, which identified ICUs as high-risk environments, with MRSA frequencies ranging from 40% to 60% in certain tertiary institutions (Ali et al., 2020; Taha et al., 2022; Alanzi et al., 2024; Almutairi et al., 2024). This indicates that the MRSA rates in ICUs are still extremely high worldwide; at some facilities, they surpass 50% of S. aureus isolates (Lee and Harbarth, 2012; CDC, 2024).

Regarding culture sources, although there was no statistically significant difference between MRSA and MSSA isolation sites (p = 0.212), MRSA was more frequently recovered from blood (55.0%), skin and subcutaneous (53.8%), and abscess samples (55.1%). This non-specific distribution agrees with global patterns, reinforcing MRSA’s versatility in causing localized and systemic infections (Turner et al., 2019; Siddiqui and Koirala, 2025).

Temporal analysis revealed a statistically significant increase in MRSA prevalence across all hospital wards, with the surgical ward showing the most dramatic rise from 46.6% in 2013 to 68.3% in 2024 (OR = 1.115, 95% CI: 1.080–1.152, p < 0.001). Similarly, upward trends were noted in medical and ICU settings. These findings are concerning but not unexpected, given rising antibiotic resistance, prolonged hospitalizations, and the intensive use of medical devices in such environments (Lee and Harbarth, 2012; Sabra and Abdel-Fattah, 2012; Lee et al., 2021; Samuel et al., 2023).

Notably, our data corroborate recent reports indicating that MRSA rates—particularly hospital-associated strains—remain a significant concern in Saudi Arabia despite ongoing infection control efforts. A 2024 meta-analysis indicated regional MRSA prevalence rates of up to 42% in western regions of Saudi Arabia, including Asir, highlighting persistent challenges (Alanzi et al., 2024).

The COVID-19 pandemic may have further complicated infection control measures, as suggested by the high MRSA rates observed among COVID-19 patients in this study (62.5%). Emerging evidence suggest that COVID-19, particularly when accompanied by immunosuppressive treatments and invasive mechanical ventilation, may increase the risk of secondary MRSA infections (Pintea-Simon et al., 2024).

Strengths of this study include its large sample size, longitudinal design, and detailed analysis by clinical setting and diagnosis. However, some limitations must be acknowledged: the retrospective design limits causal inference, molecular typing of MRSA strains was not performed, and data were drawn from a single tertiary center, potentially limiting generalizability.

## Conclusion

The results of this study provide a detailed analysis of the epidemiological and microbiological characteristics of *Staphylococcus aureus* infections over 11 years, highlighting the comparative prevalence of MRSA and MSSA across diverse clinical settings and patient comorbidities. The results reveal a steadily increasing burden of MRSA, particularly within inpatient hospital wards and among vulnerable populations such as those with urinary tract infections, diabetes mellitus, COVID-19, and end-stage renal disease.

The observed rise in oxacillin resistance across all healthcare environments underscores the critical need for strengthened infection control measures and tailored antimicrobial stewardship programs. Continuous local surveillance is essential for monitoring resistance patterns, adapting empirical therapy guidelines, and preventing further dissemination of MRSA strains.

Future efforts should focus on implementing robust national infection prevention strategies, promoting rational antibiotic use, and investing in molecular epidemiology research to understand transmission dynamics. Strengthening healthcare infrastructure, particularly in high-risk hospital wards, and enhancing community-level awareness will be pivotal in controlling the growing MRSA threat in Saudi Arabia and similar regions.

## Data Availability

The raw data supporting the conclusions of this article will be made available by the authors, without undue reservation.
